# Use of Metagenomic Shotgun Sequencing Technology To Detect Foodborne Pathogens within the Microbiome of the Beef Production Chain

**DOI:** 10.1128/AEM.00078-16

**Published:** 2016-04-04

**Authors:** Xiang Yang, Noelle R. Noyes, Enrique Doster, Jennifer N. Martin, Lyndsey M. Linke, Roberta J. Magnuson, Hua Yang, Ifigenia Geornaras, Dale R. Woerner, Kenneth L. Jones, Jaime Ruiz, Christina Boucher, Paul S. Morley, Keith E. Belk

**Affiliations:** a Department of Animal Sciences, Colorado State University, Fort Collins, Colorado, USA; b Department of Clinical Sciences, Colorado State University, Fort Collins, Colorado, USA; c Department of Biochemistry and Molecular Genetics, University of Colorado Denver School of Medicine, Aurora, Colorado, USA; d Department of Computer Science, Colorado State University, Fort Collins, Colorado, USA; INRS–Institute Armand-Frappier

## Abstract

Foodborne illnesses associated with pathogenic bacteria are a global public health and economic challenge. The diversity of microorganisms (pathogenic and nonpathogenic) that exists within the food and meat industries complicates efforts to understand pathogen ecology. Further, little is known about the interaction of pathogens within the microbiome throughout the meat production chain. Here, a metagenomic approach and shotgun sequencing technology were used as tools to detect pathogenic bacteria in environmental samples collected from the same groups of cattle at different longitudinal processing steps of the beef production chain: cattle entry to feedlot, exit from feedlot, cattle transport trucks, abattoir holding pens, and the end of the fabrication system. The log read counts classified as pathogens per million reads for Salmonella enterica, Listeria monocytogenes, Escherichia coli, Staphylococcus aureus, Clostridium spp. (C. botulinum and C. perfringens), and Campylobacter spp. (C. jejuni, C. coli, and C. fetus) decreased over subsequential processing steps. Furthermore, the normalized read counts for S. enterica, E. coli, and C. botulinum were greater in the final product than at the feedlots, indicating that the proportion of these bacteria increased (the effect on absolute numbers was unknown) within the remaining microbiome. From an ecological perspective, data indicated that shotgun metagenomics can be used to evaluate not only the microbiome but also shifts in pathogen populations during beef production. Nonetheless, there were several challenges in this analysis approach, one of the main ones being the identification of the specific pathogen from which the sequence reads originated, which makes this approach impractical for use in pathogen identification for regulatory and confirmation purposes.

## INTRODUCTION

Foodborne illness is a national and global health concern. According to the Centers for Disease Control and Prevention (CDC), foodborne pathogens are responsible for >48 million illnesses, 128,000 hospitalizations, and 3,000 deaths in the United States each year (1). The global impact of foodborne illnesses is enhanced by their significant economic impact. The costs of foodborne illness extend from the direct medical costs associated with the illness to costs incurred by the industry through product recalls, loss of consumer confidence, and litigation. Recently, Scharff (2) estimated that the aggregated annual costs of foodborne illness in the United States exceed $77 million. Given the public health and economic impact of foodborne illness, it is important to study the distribution of foodborne pathogens in food production chains and develop reliable and rapid methods for foodborne pathogen detection.

The techniques and technologies used for the detection and characterization of foodborne pathogens in food products have evolved tremendously over the past several decades (3–5). Traditional methods for pathogen detection, including microscopy and culture-based analyses, although useful, are biased according to the specific culture requirements for most genera and species, and they do not assess the microbiome at the ecological level. Although they are advanced compared to classical methodologies, more modern approaches, including immunoassays and/or nucleic acid amplification, only allow for the detection of a single pathogen or a few specific pathogens at a time. Thus, even these more advanced approaches are limited in their capacity to investigate interactions between pathogens, commensal bacteria, and the environment. Further, changes in the surrounding environment cause stresses on bacterial populations, leading to the reorganization of microbial communities, which potentially affects the persistence of foodborne pathogens in the food production chain (6, 7). Therefore, it is necessary to assess the influence that entire microbial communities have on the presence of pathogens. Shotgun metagenomics, which is the study of whole-community DNA extracted directly from samples, has increasingly been used in multiple disciplines, particularly as sequencing costs decrease and output increases (8). Furthermore, compared to target amplicon metagenomics (e.g., 16S rRNA gene sequencing), shotgun metagenomics provides the potential for both higher-resolution identification of organisms (i.e., to the strain level) and the study of microbial communities from environmental samples without introduction of sequencing bias due to unequal amplification of the target gene (9).

Food-producing animals are often considered one of the major reservoirs for foodborne pathogens, and 45.5% of hospitalizations due to foodborne pathogens have been attributed to animal production (10). Several studies have provided prevalence of single or a few foodborne pathogens in samples collected from various parts of the meat/poultry production chain (11–13). However, there are insufficient data regarding the distribution and persistence of bacterial pathogens through the entire beef production chain, from feedlot entry through to packaging of the final meat product. Additionally, no published studies have quantified changes in pathogen populations in the context of the larger microbiome using a shotgun metagenomic approach. Despite this dearth of knowledge about pathogen-microbiome dynamics, government agencies, such as the Food Safety and Inspection Service (FSIS), have started to investigate the use of sequence-based methods, including metagenomics, for pathogen detection (14). In order to provide a foundation for such efforts, the goal of this study was to address this knowledge gap by demonstrating the utility of shotgun metagenomic sequencing for detecting and characterizing the distributions of major foodborne pathogens as well as their virulence factor-related genes throughout the beef production chain.

## MATERIALS AND METHODS

### Study population.

Four geographically dispersed cattle feedlots were selected to obtain samples that were representative of that sector of the cattle industry. Two feedlots (feedlots A and B) were located in northern Colorado, and two feedlots (feedlots C and D) were located in the panhandle of Texas. All four feedlots are large-scale commercial feedlots, with capacities of 98,000, 69,000, 74,000, and 73,000 heads, respectively. Two pens from each feedlot were selected for use in this study. Shortly following their arrival at the feedlot, cattle were placed into a home pen (total, 1,741 cattle; range, 150 to 281 cattle/pen), where they were housed throughout their time at the feedlot. The animals housed in these study pens consisted of healthy steers and heifers, and all cattle were subjected to routine production practices used by each feedlot for beef production. Cattle were fed a high-grain diet and shipped to abattoir facilities in Colorado and Texas after reaching target market weight (approximately 590 kg). Because the weight and age of cattle varied when they were placed in the feedlot, the duration of their stay in the feedyard varied (average, 131 days; range, 94 to 186 days).

### Sample collection.

The same groups of animals enrolled in the study were followed longitudinally from the time of entry into feedlots, to the abattoirs where they were slaughtered, and finally to the beef products that were harvested from these cattle. A composite sample of fresh feces from the pen floor, a composite soil sample from the pen floor, and a drinking water sample were collected separately (described below). These samples were collected from every pen at the time that cattle entered the feedlot (“arrival”) and at the time of shipment for slaughter (“exit”; *n* = 16 composite fecal samples, 16 composite soil samples, and 16 water samples). Composite samples of feces were created by combining fecal pats (∼30 g each) collected by hand from 12 areas along crossing diagonals of each pen (for feedlots) or from 12 randomly selected areas of each pen (for abattoir holding pens) in a sterile Whirl-Pak bag (Nasco, Fort Atkinson, WI). Composite samples of pen surface soil were collected using the same method as for feedlot feces. Cattle drinking water was collected (1-liter samples) from the water dispenser in each pen at each feedlot or abattoir holding pen. Water within the dispensers was thoroughly mixed before collection in sterile bottles.

At the time of slaughter, cattle were shipped by truck to abattoir facilities, where they were placed in holding pens. Water and composite fecal samples were obtained from these holding pens (“holding pen”) using methods described above regarding samples collected at feedlots (*n* = 8 fecal samples and *n* = 8 water samples). Sponge samples were obtained from the walls and floors of trucks used to transport cattle from the feedlots to the abattoirs. Four to 7 trucks were used to transport cattle, depending on the number of cattle for each feedlot pen, and samples collected from a representative number (i.e., ∼60%) were composited (“truck”; *n* = 8 sponge samples). Trucks were sampled using a premoistened sponge sampling device (EZ-Reach sponge samplers prehydrated with 10 ml of Dey/Engley [DE] neutralizing broth; World Bioproducts LLC, Woodinville, WA). Two internal walls, the internal side of the door, and the floor of the truck trailers were swabbed (20 back-and-forth sponging motions on each side of the sponge).

After slaughter, carcasses from the study cattle were disassembled into standard commercial beef products (“market-ready” products). Animal and pen identity were maintained throughout slaughter, processing, and disassembly. Sponges (EZ-Reach sponge samplers; World Bioproducts, LLC) were used to sample the conveyer belts used to move chuck and round cuts after the disassembly of carcasses; 1 composite sample was collected after processing each pen (*n* = 8 sponge samples). As the conveyor belts were moving during sample collection, sponges were held on the running belt for 1 min on each side. Additionally, 400 g of beef trim was collected from processed beef (before the last application of antimicrobial interventions) for each pen of cattle (*n* = 8 trim samples). Thus, a total of 88 samples were collected (arrival, 24 samples; exit, 24 samples; truck, 8 samples; holding pen, 16 samples; and market-ready, 16 samples).

### Sample processing.

All samples collected in Colorado were transferred in insulated containers to the Food Microbiology Laboratory of the Center for Meat Safety & Quality at Colorado State University, Fort Collins, CO, within 1 h of collection. Samples collected in Texas were packed on ice in insulated containers and shipped to the same lab, where they arrived within 24 to 48 h. Fecal, soil, sponge, and trim samples were immediately stored at −80°C. Water samples were concentrated by centrifugation (15,000 × *g*, 20 min, 4°C; Eppendorf model 5810 R centrifuge; Brinkmann Instruments, Inc., Hamburg, Germany), and about 5 ml containing the pellet from each sample was stored at −80°C. Samples remained at −80°C until DNA extraction.

### DNA extraction.

After thawing at room temperature, fecal or soil samples (10 g) were mixed with 30 ml of buffered peptone water (BPW) to sediment for 10 min. Supernatants, including some fecal and soil debris, were removed to a new sterile centrifuge tube and centrifuged (4,300 × *g*, 10 min, 4°C; Eppendorf model 5810 R centrifuge). The pellet from each sample was rinsed with 5 ml of molecular-grade sterile phosphate-buffered saline (PBS) and centrifuged again (4,300 × *g*, 10 min, 4°C). The supernatant was removed, and the resulting pellet was resuspended in 15 ml of PowerBead solution (Mo Bio Laboratories, Inc., Solana Beach, CA). DNA extraction of the fecal and soil samples was performed using the Mo Bio PowerMax soil DNA isolation kit (Mo Bio Laboratories, Inc.), according to the manufacturer's protocol.

Thawed meat trimmings (400 g) were rinsed with 90 ml of BPW. After storage at 4°C to solidify fat, the liquid content of the rinsate was removed and centrifuged (4,300 × *g*, 20 min, 4°C). Pellets were resuspended in 5 ml of cold sterile saline solution (0.85% NaCl in sterile water). The cold saline wash was centrifuged (4,300 × *g*, 20 min, 4°C), and 250 mg of the resulting pellet was utilized for DNA extraction. For sponge samples, the sample liquid was removed from the sponge by hand squeezing into the bag, and the liquid was pipetted to a collection tube. Following the initial squeezing extraction, 10 ml of BPW was added to the sponge, and squeezing was repeated. The liquids from each extraction were combined and centrifuged (4,300 × *g*, 20 min, 4°C). Pellets from all truck sponge samples for one pen of cattle were combined, as were pellets from the abattoir fabrication room (round, chuck, and trim conveyor belts) for one pen of cattle. The combined samples were then centrifuged (4,300 × *g*, 20 min, 4°C), and the pellet was collected for DNA extraction. DNA was extracted from 250 mg each of water, composite sponge, and trimming rinsate pellet using the Mo Bio PowerFecal DNA isolation kit (Mo Bio Laboratories, Inc.), according to the manufacturer's protocol.

Extracted DNA from fecal and soil samples was eluted in 5 ml of the kit elution buffer, and water, sponge, and trimming rinsate samples were eluted in 50 μl of the kit elution buffer. DNA concentrations were measured at 260 nm using a NanoDrop spectrophotometer (Thermo Fisher Scientific, Inc., Pittsburgh, PA). Samples with concentrations of <20 ng/μl were concentrated using standardized ethanol precipitation techniques.

### DNA library preparation and sequencing.

After DNA extraction, 100 μl of DNA of each fecal and soil sample and 30 μl of DNA of each water, sponge, and trim rinsate sample were delivered to the Genomics and Microarray Core at the University of Colorado Denver (Aurora, CO) for metagenomic sequencing. Sample libraries were constructed using the Illumina TruSeq DNA library kit (Illumina, Inc., San Diego, CA) for samples that contained at least 1 μg of DNA and using the Ovation Ultralow DR multiplex system 1–8 and 9–16 (NuGEN Technologies, Inc., San Carlos, CA) for samples that contained <50 ng of DNA, according to the manufacturer's protocols. Library sequencing (paired-end, 2× 100 bp) was performed on the Illumina HiSeq 2000 (Illumina, Inc.).

### Bioinformatics analysis.

The sequence data were first trimmed and filtered using Trimmomatic (15). Adapters supplied in the Illumina TruSeq3 adapter sequence file were removed by Trimmomatic's ILLUMINACLIP command. Next, the first three and last three nucleotides were removed from each read, and a sliding window of four nucleotides was checked based on average Phred score (Qscore). Nucleotides within these windows were removed until the average Qscore across the window was >15. Finally, the sequence reads with <36 bp were removed, along with their mate-pair reads.

The remaining sequence reads were aligned to the host reference genome (Bos taurus UMD3.1) using Burrows-Wheeler aligner (BWA) (16), with default settings in a paired-end manner. The reads that aligned to B. taurus were removed from further analysis. Nonhost reads were then classified using Kraken (17) for both pathogen identification and microbial taxonomy analysis. The number of reads classified to each taxon was recorded as the raw count. In addition, the nonhost reads were aligned to a modified Virulence Factor Database (VFDB) using BWA at default settings in a paired-end manner (18). Redundant sequences (100% identical sequences and reverse complement sequences) were removed from a combined R1 and R3 VFDB database using CD-HIT. The results (SAM file) created by BWA were parsed using a custom-developed Java-based script to calculate the gene fraction for each virulence factor (VF)-related gene in each sample. An 80% gene fraction threshold (i.e., 80% of the full length of each VF-related gene had to be covered by at least one read assigned to that gene within each sample) was applied arbitrarily to identify potential positive VF-related genes in the sample.

### Statistical analysis.

The major foodborne pathogens investigated in the analysis were Salmonella enterica, Listeria monocytogenes, generic Escherichia coli (as a marker for pathogenic enteric bacteria), Staphylococcus aureus (as a marker for toxigenic strains), Clostridium spp. (C. botulinum and C. perfringens), and Campylobacter spp. (C. jejuni, C. coli, and C. fetus).

In order to investigate potential shifts in abundance for pathogen composition in samples collected at different sites and times in the production process, counts per million reads were calculated using raw counts assigned to each pathogen at the species level (sum of counts of C. botulinum and C. perfringens for Clostridium spp. and sum of counts of C. jejuni, C. coli, and C. fetus for Campylobacter spp.) divided by the total number of trimmed and filtered reads of the sample multiplied by one million. In order to further understand how pathogens (at the species level) change within the microbiome, raw counts were quantile normalized (raw counts divided by the total number of mapped reads to all bacteria within each sample and multiplied by a normalization scale factor based on count shift distribution within the sample) using the metagenomeSeq R package (19). The normalized counts were reported at the phylum and species levels. As an internal assessment, changes in the normalized counts of two bacteria, Selenomonas ruminantium and Pseudomonas fluorescens, were also assessed.

Shannon's diversity index at the species level was calculated for each sample using the vegan R package (20). Analysis of variance (ANOVA) tests were performed using SAS version 9.3 (SAS Institute, Inc., Cary, NC), using the proc mixed function to test for site/time effects on log counts per million reads and Shannon's diversity index of samples. Pairwise comparisons of the log fold change of normalized counts were tested using zero-inflated Gaussian mixture models within metagenomeSeq's fitZig function and limma's makeContrasts function (21). Nonmetric multidimensional scaling (NMDS) ordination using Euclidean distances on Hellinger-transformed normalized counts for bacterial species was calculated and followed by the anosim function for analysis of similarities by site/time or sample matrix using the vegan R package (20, 22). The results from anosim were reported as R values, along with *P* values and stress values (23). In all the models, site/time was considered the main fixed variable of interest, and pen was considered to be the experiment unit for repeated measures. An α value of 0.05 was used to determine statistical significance for all analyses.

### Nucleotide sequence accession number.

Raw sequencing reads for all 87 samples described in this project have been deposited in the NCBI Sequence Read Archive under accession no. PRJNA292471.

## RESULTS AND DISCUSSION

### Sequence data.

A total of 87 samples were sequenced successfully. (One of the drinking water samples did not yield sufficient DNA for sequencing.) The average number of reads per sample was 46.3 million (range, 12.0 million to 93.4 million reads/sample). The average Phred score was 35.2 (range, 33.7 to 36.3). Across all samples, 89.9% of the base calls had an average Phred score of ≥Q30. An average of 5.1% of reads were removed from the sample data by Trimmomatic (see Table S1 in the supplemental material).

### Classification level.

Before our data were analyzed, a test was performed to evaluate the consequences of using Kraken as a classifier when considering genomic sequences of bacteria whose reference genomes are not included in the NCBI RefSeq database used by Kraken (17). We were interested in both the sensitivity and specificity of these classifications, considering the significant consequences regarding food safety and the regulatory consequences relative to false-positive and false-negative classification results. Hence, using a published sequence for S. enterica serovar Cerro (K serogroup, GenBank accession no. NZ_AOZJ00000000.1), we simulated error-free 125-bp sequence reads and then classified these reads using Kraken (17). The Salmonella serovar Cerro genome was chosen, because the reference genome for this serovar is not present in the NCBI Ref database, which was used by Kraken at the time of our analysis. Our results demonstrated that >90% of the simulated reads were correctly classified at the species level (S. enterica). However, the remaining reads were distributed as matches to several other Salmonella serovars that were present only in the Kraken database (e.g., S. enterica serovar Typhi, S. enterica serovar Newport, etc.), as well as other species in the phylum Proteobacteria. Based on these results, the decision was made to use Kraken classification results from only the species level or above and not at the serovar or strain level. Accordingly, we also are only reporting data for E. coli at the species level instead of attempting to identify and differentiate various strains of pathogenic E. coli, such as O157:H7 or other non-O157 Shiga toxin-producing E. coli (STEC).

### Shifts in pathogen abundance.

Kraken's basic algorithm is to perform a massive comparison of all genomes in the NCBI RefSeq database in order to identify uniquely identifying sequences (or k-mers) for each organism and taxon. These unique sequences can then be used to taxonomically classify reads within a metagenomic sample. While extremely rapid and relatively accurate, this approach introduces some bias into calculations of abundance. Namely, pathogens with genomes that contain comparatively higher numbers of unique k-mers may receive more hits than pathogens with comparatively lower numbers of unique k-mers, despite being present in equal abundance. Therefore, it is not valid to compare the abundance of different bacteria within a sample or group of samples. However, since the bias for each specific bacterium remains constant across samples, it is valid to compare differences in abundances of individual agents between samples or sample groups. Therefore, currently, it is difficult to quantify the absolute numbers of pathogen cells from environmental samples using the shotgun metagenomic approach and available bioinformatics tools.

**FIG 1:**
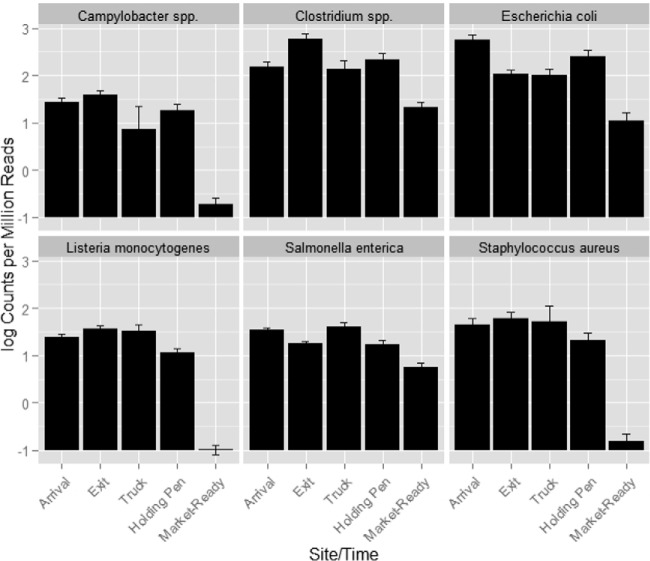
Least-square means with standard error of log read counts per million reads of each investigated pathogen/bacterium from samples collected at different sites/times (arrival, *n* = 24; exit, *n* = 24; truck, *n* = 8; holding pen, *n* = 15; and market-ready samples, *n* = 16).

While the patterns of change in abundance across all sampling sites/times were quite different among the microbial agents investigated, all six pathogen groups decreased dramatically in log read counts per million reads from the arrival samples to the market-ready samples (Fig. 1, *P* < 0.05). These results support the efficacy of current food safety interventions, such as knife trimming, steam vacuuming, hot-water pasteurization, organic acid sprays, and chilling, all of which have been widely demonstrated to effectively reduce pathogens and total bacterial load (24–28). For example, Wheeler et al. (29) summarized a 1- to 4-log CFU reduction(s) in microbial populations due to commonly utilized antimicrobial interventions to the surface of beef carcasses or subprimals, indicating that pathogens and nonpathogenic bacteria are both effectively targeted by these interventions.

**FIG 2:**
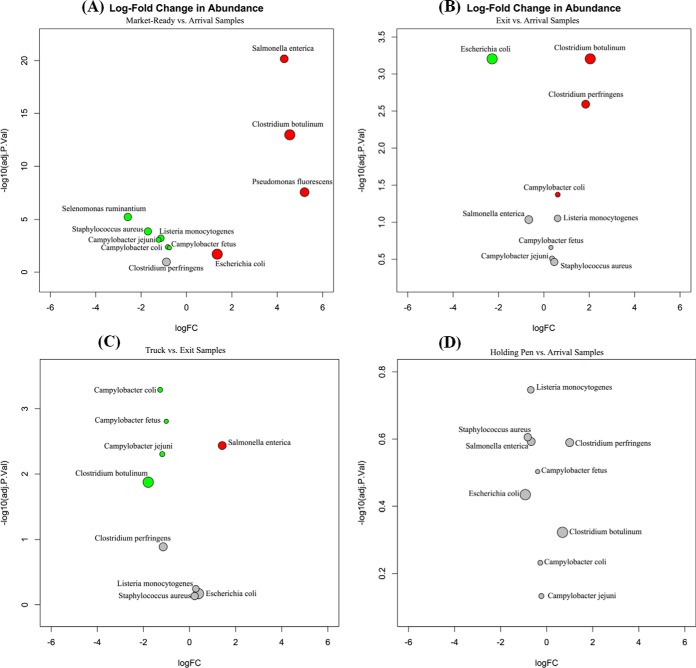
(A to D) Pairwise comparison of log fold change (logFC) of normalized counts of investigated pathogens and nonpathogens between market-ready and arrival samples (A), exit and arrival samples (B), truck and exit samples (C), and holding pen and arrival samples (D). Red circles indicate a significant (adjusted *P* value [adj.P.Val] < 0.05) increase in normalized counts of bacterial species in samples collected at a former site/time, and green circles illustrate a significant (adjusted *P* < 0.05) decrease in normalized counts of bacterial species in samples collected at a former site/time within comparisons. Gray circles indicate that the change between samples collected at different sites was not significant. The size of the circles is proportional to average normalized counts of corresponding bacterial species across all samples.

A challenge when analyzing data from this study was the proportion of sequence reads in some samples that were aligned to B. taurus. Specifically, in market-ready samples, >99% of reads were classified as B. taurus. This result is logical considering the source of these samples (sponge samples from conveyer belts and rinsates of meat) and the fact that 90% of bacteria were killed or removed during the slaughtering process. Because of this, there also is a large difference between these sample matrices in the proportion of reads belonging to the bacterial microbiome relative to the total reads. To address this issue, we employed quantile normalization to investigate the change in proportion of these pathogens within the total microbiome, and we adjusted the sequence depth based on the distribution of counts that were assigned to all bacteria within each sample. Pairwise comparisons of the log fold change of normalized counts were made between market-ready and arrival samples, exit and arrival samples, truck and exit samples, and holding pen and exit samples (Fig. 2A to D). This analysis revealed that while the log read counts per million reads decreased dramatically for all 6 pathogens, the normalized counts of S. enterica, C. botulinum, and generic E. coli were higher (adjusted *P* < 0.05) in the market-ready samples than those in the arrival samples. This suggests that although the overall abundances of E. coli, S. enterica, and C. botulinum were reduced by postharvest interventions, the proportions of these pathogens within the whole microbiome increased from the arrival to market-ready samples.

It is known that some bacteria, including pathogens, may survive interventions and persist in beef production (7, 30). For example, the endospore produced by C. botulinum is thermoduric (31). Furthermore, although interventions are useful in the mitigation of surface bacteria, their efficacy may be limited against internalized pathogens. In food animals, harborage of Salmonella in peripheral lymph nodes has been observed (32); in this manner, pathogens are protected from surface-based antimicrobial interventions and can then be introduced into the processing environment during disassembly of the beef carcass. Since many other surface pathogens and bacteria are eliminated or drastically reduced during the application of surface antimicrobial treatments, Salmonella spp. from broken lymph nodes that are exteriorized or opened during fabrication could easily become the predominant bacteria within the remaining microbiome. The unequal efficacy of antimicrobial interventions against pathogenic bacteria, either due to internalization or cross-protection, provides a scenario in which the diversity of the microbiome, although shrinking, may result in a higher relative abundance of C. botulinum and S. enterica.

Two indicator bacterial species were used to validate our results, S. ruminantium and P. fluorescens (Fig. 2A). S. ruminantium is a primary rumen bacterium and should not be prevalent on meat samples if meat products are not contaminated by fecal material (33). Therefore, the decrease in normalized counts for S. ruminantium in market-ready samples compared to that in arrival samples was expected. Conversely, P. fluorescens is one of the predominant spoilage bacteria associated with beef, due to its proclivity for low-oxygen environments (34). It is commonly found on beef tissues, and therefore, its increase in relative abundance in market-ready samples was also anticipated. The observed changes in these bacteria provided support for the validity of results regarding the changes in abundance of normalized counts for pathogens.

When examining comparisons between the arrival and exit samples, the normalized counts for the majority of the investigated pathogens did not change significantly (*P* > 0.05). Of those with observable changes, the two Clostridium spp. increased, while those of generic E. coli declined significantly. Similarly, no significant change in normalized counts for any investigated pathogens was observed between holding pen and arrival samples. Although season of the year was not incorporated into the statistical model or sampling design, anecdotal observations of sample collection periods may provide some useful insight into shifts in the microbiome. For example, exit and holding pen samples were collected in colder seasons (late November to late January), while all of the arrival samples were collected in the hot season (middle of July to early September).

### Microbiome composition.

**FIG 3:**
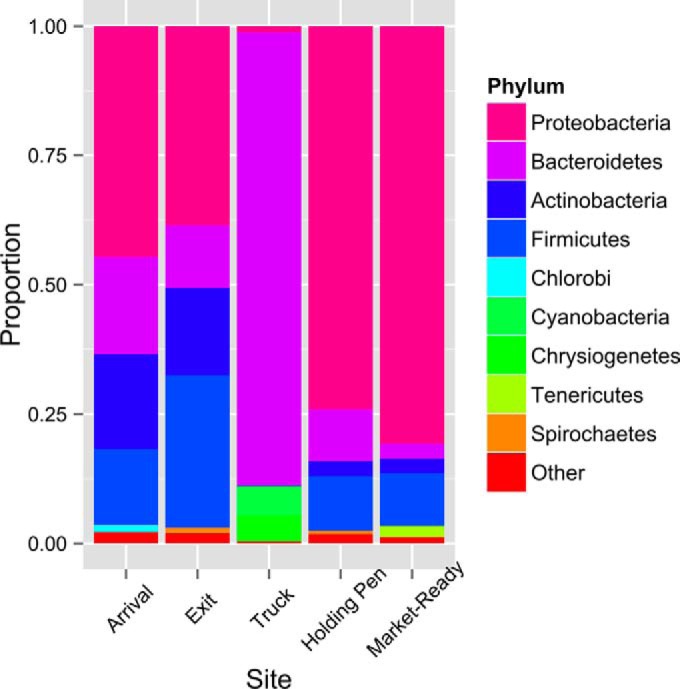
Microbiome composition at the phylum level for samples collected at different sites/times. The top 5 phyla (accounts for >97% of matches at the phylum level) were reported for each site/time, and all other phyla were grouped into “Other” for each site/time.

The five primary phyla (accounting for >97% of the aligned reads) for samples collected at each sector of the beef production system are shown in Fig. 3. The predominant phylum for the arrival, exit, holding pen, and market-ready samples was Proteobacteria, followed by Actinobacteria, Firmicutes, and Bacteroidetes; however, the proportions of Actinobacteria, Firmicutes, and Bacteroidetes varied by sampling site/time. The proportion of Bacteroidetes was extremely high (∼87.5%) in truck samples. The other major phyla identified in truck samples were Cyanobacteria and Chrysiogenetes, which were not commonly identified in the other study samples. The composition of bacteria at the phylum level provided an overview of the microbiome of samples; however, it was not helpful in understanding the change in the abundance of pathogens, because pathogen information is difficult to retrieve at the phylum level. Thus, analysis of the microbiome at a higher resolution (species level) was performed.

**FIG 4:**
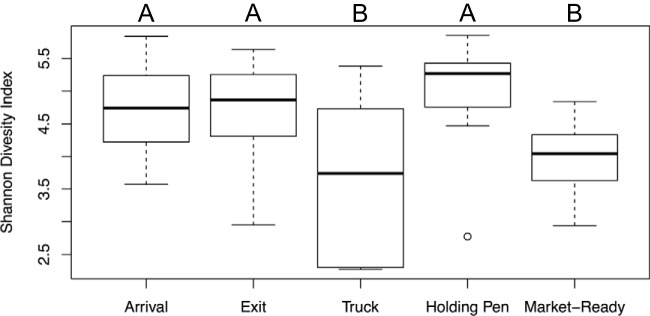
Box plot of Shannon's diversity index for samples collected at different sites/times (arrival, *n* = 24; exit, *n* = 24; truck, *n* = 8; holding pen, *n* = 15; and market-ready samples, *n* = 16). Different letters (A and B) indicate that the least-squares means of Shannon's diversity index differed among samples collected at different sites/times (*P* < 0.05). The circle indicates an outlier.

Among all samples, a total of 1,317 bacterial species were identified by Kraken. Shannon's diversity index at the species level did not differ significantly (*P* > 0.05) between arrival, exit, and holding pen samples but was lower (*P* < 0.05) in both truck and market-ready samples (Fig. 4). The apparent similarity in the abundance of pathogens in the arrival and exit samples may be due to homeostasis of the microbiome in the feedlot environment. Likewise, the absence of detectable changes in the proportion of pathogen groups in the arrival and holding pen samples was perhaps due to the similar microbiome diversity of these two groups of samples. The reduced microbiome diversity for market-ready samples supports the notion that the decrease in read counts per million reads for pathogens in market-ready samples stemmed from the effect of antibacterial interventions applied in the beef abattoir, although the decreased microbiome diversity could also be attributed to low sequence depth on the bacteria in these samples (35).

**FIG 5:**
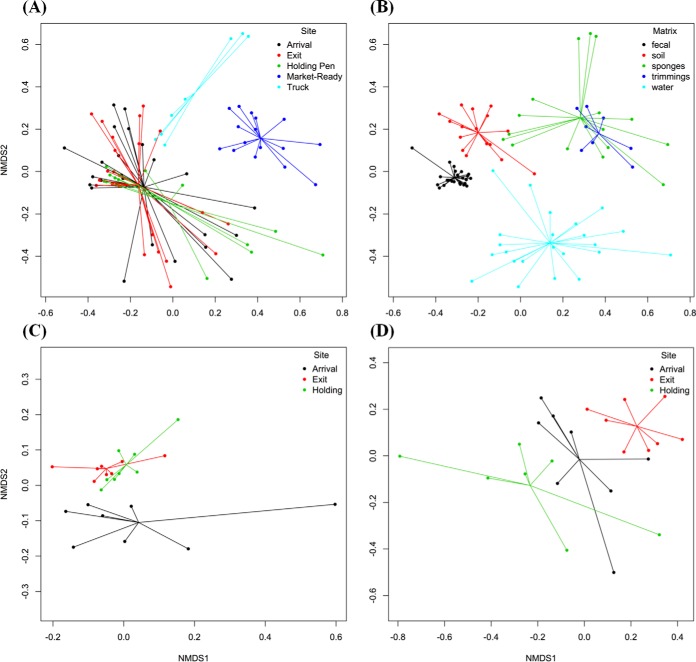
Nonmetric multidimensional scaling (NMDS) for ordination plots of normalized counts at the species level. The results of the analysis of similarities (anosim) for them were *R* = 0.3888, *P* = 0.001, stress = 0.166, by site/time (A); *R* = 0.7217, *P* = 0.001, stress = 0.166, by matrix (B); *R* = 0.2128, *P* = 0.001, stress = 0.105, by site/time (for fecal samples only) (C); and *R* = 0.4143, *P* = 0.001, stress = 0.131, by site (for water samples only) (D).

Nonmetric multidimensional scaling (NMDS) ordination revealed that the arrival, exit, and holding pen samples clustered separately from the truck and market-ready samples (Fig. 5A to D). This suggests that sampling site/time played an important role in microbiome changes within each sector of the beef production chain. However, sample matrix was a confounding factor, since sponge and meat rinsate samples were collected from trucks and beef abattoirs, while fecal, water, and soil samples were collected at feedlots and holding pens. The NMDS ordination supported sample separation by matrix (*R* = 0.722, *P* = 0.001, stress = 0.166). While different sample matrices contributed to microbiome divergence at each step of the beef production chain, it is important to note that these sample matrices reflect the nature of the environment at each sector of the beef production chain; for instance, feces, soil, and water are the primary components of the beef feedlot environment, while the truck interior surfaces are the primary environment to which cattle are exposed during transport. In order to avoid confounding by sample matrix, NMDS ordination and the anosim function of vegan were performed separately for fecal and water samples collected at arrival, exit, and holding pen. Within matrix, bacterial composition differed significantly (*P* = 0.001) between arrival, exit, and holding pen samples (Fig. 5C and D). However, the *R* values for these comparisons were 0.2128 for the site comparison within fecal samples and 0.4143 within water samples, indicating that the degree of separation by site was not distinguishable. Therefore, the lack of a large microbiome shift between arrival, exit, and holding pen samples may have contributed to the relative stability in the proportion of investigated pathogens within the microbiome in these environmental samples.

### Virulence factors.

**FIG 6:**
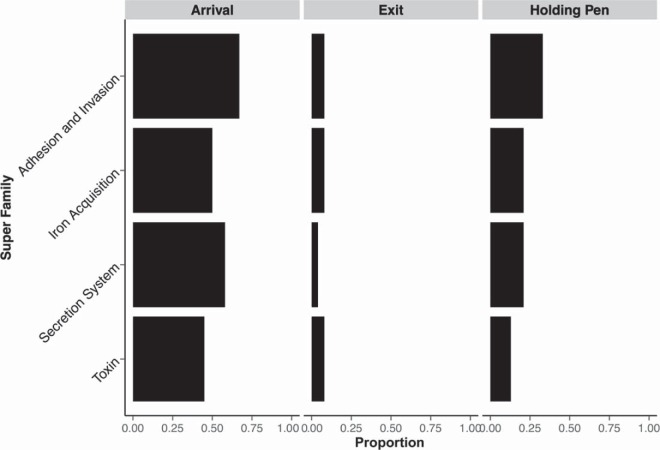
Proportion of arrival (*n* = 24), exit (*n* = 24), and holding pen (*n*= 15) samples that contained at least one virulence factor from four superfamilies.

A total of 76,254 reads were assigned to 1,383 VF-related genes (63 VFs) from 28 samples. These VFs belonged to four (out of seven) superfamilies, namely, adhesion and invasion, secretion systems, toxins, and iron acquisition. The proportions of samples collected at arrival, exit, and holding pen sites, which contained at least one VF (by VF superfamily), are shown in Fig. 6. Only one VF was identified in one truck sample, and no VFs were identified in the market-ready samples. The majority of the arrival samples contained VFs of the four superfamilies. Notably, VFs were detected in only two exit samples.

Interestingly, 233 VF-related genes that were identified in arrival but not in exit samples were assigned to E. coli in VFDB. To confirm the specificity of these VFs to E. coli, we performed a BLAST search of the NCBI nonredundant/nucleotide (nr/nt) collection database on all of these VFs, using default settings. Based on this search, over half of these VF-related genes were indeed specific to E. coli species, a finding that corroborates the decrease in E. coli read counts per million reads observed between the exit and arrival samples. Together, these results suggest that feedlot management practices effectively control generic E. coli during the feeding period.

The primary VFs identified in three holding pen samples from the same geographic region (i.e., TX) are purported to originate solely from Aeromonas hydrophila and Aeromonas salmonicida, waterborne pathogens of humans and salmonid fish, respectively (36, 37). Interestingly, an extremely large number of reads (range, 1.36% to 2.38% of total filtered reads) were classified under these two bacterial species within these same three holding pen samples, suggesting that there may have been contamination in the holding pen water.

### Assessment of the metagenomic approach.

Although shotgun metagenomics provides a unique lens for viewing the microbiome, there are important limitations regarding the identification and characterization of specific agents, such as foodborne pathogens. Whole-genome sequences have enabled the generation of high-quality reference genomes that can be used for bacterial identification by analyzing the alignment of sequence reads. However, there are some limitations in aligning metagenomic data to the reference genomes of bacteria. First, conserved genomic regions are always present among bacteria. For instance, a read (∼100 bp) that has been assigned to the Salmonella serovar Newport reference genome could have indeed originated from a Salmonella serotype Newport bacterium; however, it could also have come from Salmonella Typhimurium or another Enterobacteriaceae, such as E. coli, whose genome shares the same conserved DNA fragment. Therefore, reads that align to any of these conserved regions of a pathogen cannot be used for differentiation. In addition, within an environmental sample containing a highly diverse bacterial community (e.g., soil sample), only a very small proportion of reads can be assigned to known bacteria, and an even smaller proportion will be assigned to pathogens. In addition, at the sequencing depth in this study, on average <2% of the S. enterica genome and <0.5% of the L. monocytogenes genome, were covered across all samples, as determined using BWA with default parameters.

As an alternative to recovering the full-genome sequence of pathogens from shotgun metagenomic data, it may be useful to identify pathogen-specific genetic sequences as a proxy for pathogen identification, such as those that are used for PCR. The presence of VF-related genes (average length, <2,000 bp) has been detected by PCR to identify pathogens in enriched samples (38, 39). Our study demonstrated the utility of this approach for the identification of VF-related genes in diverse microbial communities. A high identity threshold (i.e., BWA default) combined with a gene fraction (80%) lends confidence to the identification of VF genes within metagenomic data. However, this approach has significant limitations when considering agents that are nonpathogenic in the absence of specific VFs, such as the case for strains of E. coli O157:H7, which are only pathogenic if they contain genes for *eae* and *stx*. Using metagenomic data, it is not possible to definitively link VF-related genes with specific pathogens. Horizontal gene transfer adds further complexity to this issue, as this mechanism can allow VF-related genes to be found in a variety of pathogenic and nonpathogenic bacteria (40). For example, one VF-related gene (chemotaxis methyltransferase, VF identification [VFID] Z2938) originally identified in E. coli O157:H7 strain EDL933 is also found in other pathogenic E. coli O157:H7 strains, such as SS52, SS17, and EC4115. Even more troubling, the same VF-related gene is also 100% homologous to a segment of DNA from the whole-genome sequence of the nonpathogenic E. coli K-12 strain. Moreover, a Shiga toxin gene (VFID VFG2056) identified in many of the samples in this project was originally isolated from Shigella dysenteriae Sd197 (serotype 1) but shares 100% sequence homology with E. coli strain SWUN4027 Stx 1 holotoxin A subunit and Stx 1 holotoxin B subunit genes (41). In addition, it may be necessary to require the presence of more than one VF-related gene in a bacterial cell to determine its pathogenicity/virulence (i.e., both *eae* and *stx* genes are required to be present in a PCR sample to confirm the presence of pathogenic E. coli). However, even though all necessary VF genes for one pathogen have been detected in samples, the shotgun metagenomic approach has no capacity to identify if these VF related genes are from one cell or from multiple cells (i.e., different bacterial species). In addition, without further transcriptomic and/or proteomic analyses, the expression of detected virulence genes is undetermined. Hence, VF-related gene identification should not be used as a single indicator of pathogenicity.

Alternatively, the utilization of genomic regions that are known to be unique and specific to particular pathogens may be a better method for pathogen detection in metagenomic samples. In theory, unique regions can be identified by comparing all the reference sequences in a given database. However, ensuring that such regions are truly specific to particular pathogens depends entirely on the size and coverage of the database. Thus, an unknown pathogen or a pathogen that lacks a reference genome in the database but that shares highly homology with an existing reference genome is likely to be misclassified, as we demonstrated using the genome from S. enterica serovar Cerro. Although a database containing as many bacterial sequences as possible is preferred, the quality of included sequences cannot be compromised. Many bacterial whole-genome sequences have been submitted to NCBI, but their accuracy is varied, and any contamination in the sequence data in the database can cause sequence misclassification (42). Therefore, when a single read is assigned to a putatively unique region of a pathogen, it does not necessarily mean that the pathogen is present in the sample. These challenges emphasize the criticality of improving the management of bacterial whole-genome sequence databases for the long term.

### Conclusions.

Our results characterize the first longitudinal metagenomic study of changes in the abundance of specific bacterial pathogens through the beef production system. This investigation differs from studies using traditional culture-based methods, as shotgun metagenomic methods allow us to investigate the proportional change of pathogens within the larger microbiome. The relative abundance of both nonpathogenic bacteria and pathogens of interest was reduced dramatically from that in samples collected in the feedlot to the final meat products. The use of standard antimicrobial interventions in the beef processing system significantly reduced the diversity of the remaining microbiome. However, the relative proportions of some of the bacteria/pathogens (E. coli, C. botulinum, and S. enterica) in the remaining microbial community were increased, potentially due to their hardy nature (e.g., C. botulinum) or their ability to be remain hidden from the antimicrobial treatments (e.g., S. enterica). In addition, the increase in the proportion of C. botulinum and Salmonella spp. in the context of an overall dramatic decrease in microbiome diversity suggests that these pathogens may be recovered in increased relative abundance due to decreased competition from other bacteria within the microbiome present on the final meat products.

While we believe the metagenomic approach has great utility for investigating the ecology of foodborne pathogens, it is important to note that metagenomic methods cannot currently be used for identification and quantification of pathogens for regulatory purposes due to limitations of the currently available technology and the incompleteness of bacterial genome databases. Specifically, the misclassification that is inherent to the read length, the inability to get deep coverage of the pathogenic organisms in the sample due to the existence of other prokaryote and eukaryote DNA within the sample, and the impossibility of obtaining a comprehensive database containing all possible pathogenic organisms of interest invalidates the use of this approach for regulatory purposes. This is especially true given the high demand for specificity in tests used for regulatory surveillance. Overall, our results strongly indicate that the shotgun metagenomic approach is not yet practically ready for pathogen identification for regulatory purposes. However, given appropriate sequencing depth, shotgun metagenomics could be utilized as a screening tool to provide an overview of pathogens, virulence factors, and antimicrobial resistance genes potentially present in an environmental sample. Culture-based methods with increased sensitivity, followed by subsequent whole-genome sequencing, may be an alternative approach for foodborne pathogen confirmation/tracking.

## Supplementary Material

Supplemental material
