# Supplementary material for: Use of Metagenomic Shotgun Sequencing Technology To Detect Foodborne Pathogens within the Microbiome of the Beef Production Chain
Source: Appl Environ Microbiol. 2016 Apr 4;82(8):2433–43. doi: 10.1128/AEM.00078-16 (PMC4959480; doi:10.1128/AEM.00078-16)
Supplement: Supplemental material [file supp_82_8_2433__index.html]

Use of Metagenomic Shotgun Sequencing Technology To Detect Foodborne Pathogens within the Microbiome of the Beef Production Chain — Supplemental material 

# Use of Metagenomic Shotgun Sequencing Technology To Detect Foodborne Pathogens within the Microbiome of the Beef Production Chain

## Supplemental material

- Supplemental file 1 -

  Sample metadata and read statistics (Table S1).

  XLSX, 19K
